# The Safety of The Directly Acting Antiviral Treatment For Hepatitis C Virus According To The Egyptian National Program Protocol In Patients With Midrange Ejection Fraction

**DOI:** 10.5334/gh.906

**Published:** 2021-01-04

**Authors:** Ahmed Farouk Alaarag, Ahmed Mohamed Hamam, Osama Ahmed Amin

**Affiliations:** 1 Department of Cardiology Tanta University, EG; 2 Department of Internal Medicine, Armed Forces College of Medicine, EG; 3 Department of Cardiology, Beni Suef University, EG

**Keywords:** Hepatitis C Virus, Directly Acting Antiviral Agents, Midrange Left Ventricular Ejection Fraction

## Abstract

**Background:**

The Egyptian National Committee of Viral Hepatitis program is the leading national hepatitis C virus (HCV) management program globally. However, limited data is available about the effect of the new directly acting antiviral agents on the cardiovascular system.

**Objectives:**

Our study aimed to assess the safety of the relatively new directly acting antiviral agents approved by the National Health Committee in Egypt to treat patients infected with hepatitis C virus who have midrange left ventricular ejection fraction.

**Methods:**

This multicenter study included 400 successive patients with an ejection fraction (40–49%) from May 2017 to December 2019. We classified them into two groups: Group I (Child A), who received Sofosbuvir and Daclatasvir for twelve weeks, and Group II (Child B), who received Sofosbuvir, Daclatasvir, and Ribavirin for twelve weeks. Patients were evaluated for their symptoms, ejection fraction, brain natriuretic peptide, lipid profile, fasting blood glucose, fasting insulin, Homeostatic Model Assessment of Insulin Resistance levels, and Holter monitoring (just before the start of treatment and within three days after completing therapy).

**Results:**

We found New York Heart Association Class, ejection fraction, brain natriuretic peptide, premature ventricular contractions burden, as well as highest and lowest heart rate did not show a statistically significant difference in both groups after treatment. The treatment did not cause bradycardia or non-sustained ventricular tachycardia. Fasting blood glucose and fasting insulin levels declined, with improved insulin resistance after treatment in both groups. Both low and high-density lipoprotein cholesterol increased after treatment in Group II.

**Conclusions:**

Both regimens of directly acting antiviral agents used in Egypt to treat chronic hepatitis C virus infection are safe in patients with New York Heart Association Class I and II with midrange left ventricular ejection fraction (40–49%). There are beneficial metabolic changes following HCV clearance as an improvement of insulin resistance.

## Introduction

Hepatitis C virus (HCV) is a single-stranded ribonucleic acid (RNA) virus. HCV has a worldwide prevalence of 2.5% and infects 180 million people worldwide [1]. HCV infection in Egyptian people has high health, economic, and social burden. Egypt had the highest HCV prevalence globally. According to the Egyptian Demographic Health Survey (EDHS) in 2008, the prevalence rate of HCV infection in Egypt was 14.7% in the 15–59 year age group [2].

The Egyptian National Committee for Control of Viral Hepatitis (NCCVH) program is the world’s leading national HCV program. This screening and mass treatment program put Egypt on a fast track to HCV elimination, making it potentially the first country to achieve the WHO disease elimination targets [3].

Chronic HCV infection has been linked to subclinical and clinical cardiovascular diseases (CVD). The proposed mechanisms include chronic inflammation and immune activation driven by HCV infection as well as direct endothelial invasion and dysfunction, but no mechanism could be confirmed till now [1,4].

Chronic HCV infection interferes with glucose and lipid metabolism, resulting in insulin resistance and diabetes mellitus (DM). The potential mechanism is to interfere with insulin signaling pathways related to increased tumor necrosis factor (TNF-α) and higher HCV viral load [1]. Also, HCV replication within liver cells may impact insulin and lipid metabolism. The changes in both can affect cardiac function by their effect on atherosclerosis [5,6]. Furthermore, interferon (IFN) and the directly acting antiviral agents (DAAs) therapies significantly improved cardiovascular outcomes. However, limited data is available about the effect of the new DAAs on the cardiovascular system after virus eradication [7].

The patients with myocardial dysfunction were unable to tolerate IFN. The novel DAAs do not have the harmful effects of IFN on heart performance. Therefore, the relatively new drugs may offer a better chance for such patients to receive antiviral therapy [8].

There is no robust data for the safety of the novel DAAs used to treat chronic HCV with other comorbidities [8]. The lack of published evidence-based data on these agents’ safety in patients with cardiac dysfunction compelled the need for this study. Patients with heart failure and midrange ejection were selected to provide evidence on the safety of novel DAAs in this group of patients. The treating physicians excluded most patients with advanced heart failure due to shorter life expectancy and lack of safety evidence.

## Methods

In this prospective multicenter study, we enrolled 400 chronic HCV patients with midrange LVEF (40% to 49%) [9] from May 2017 to December 2019. We selected them during the routine workup for HCV treatment according to the NCCVH protocol. All study patients signed written informed consent. The local research committees approved the study following the Declaration of Helsinki. The Committees of Research and Medical Ethics at Tanta University, Beni Suef University, and Kobry El Kobba Hospital approved the protocol with approval reference numbers 08/17, C52017, and 7-04/2017, respectively.

We included chronic HCV patients in our cohort cross-sectional study. All included patients were eligible for receiving treatment according to the NCCVH protocol for HCV with EF (40–49%) and NYHA functional Class I or II. All patients were taking standard evidence-based anti-heart failure treatment.

We categorized the patients into two groups based on the appropriate DAAs regimens for their Child-Pugh Class [10]. Group I (Child A) included 200 successive patients who were assigned to receive Sofosbuvir and Daclatasvir (SOF+ DCV) for twelve weeks. While, Group II (Child B) included 200 successive patients who were assigned to receive Sofosbuvir, Daclatasvir, and Ribavirin (SOF+DCV+ RBV) for twelve weeks. It is to be noted that five patients in Group I (2.5%) and three in Group II (1.5 %) were on statin therapy, which was stopped prior to initiation of antiviral therapy.

We excluded from our study any patients with advanced liver cirrhosis (bleeding esophageal varices, more than mild ascites on abdominal ultrasound or hepatic encephalopathy), autoimmune hepatitis, renal failure, diabetes mellitus, pregnancy, NYHA class III and IV or EF <40%, patients taking amiodarone, patients with bradycardia (heart rate below 60) and any patient who did not attend the follow-up visit.

The patients were assessed just before starting the treatment, at the follow-up visit within three days after the end of treatment, and at confirmation of virus clearance (after three months). We evaluated the patients’ heart failure state by NYHA Class [11] and BNP levels. We also assessed EF using the M mode by the Teicholz formula. 2D mode by the biplane Simpson method was utilized when there were regional wall motion abnormalities (RWMAs) [12]. To reduce the inter-observer variability, the average of two operators’ measures of the EF were used. Additionally, the cardiologists doing echocardiography during the follow-up did not know the previous EF before starting treatment. We performed resting ECG and Holter monitoring for 24 hours. The laboratory assessment included aspartate amino transferase (AST), alanine amino transferase (ALT), serum bilirubin, serum albumin, prothrombin time (PT), international normalized ratio (INR), and HCV RNA by polymerase chain reaction (PCR). Further, patients’ metabolic profile were assessed by serum creatinine, lipid profile, 8-hour fasting blood glucose (FBG) level, 8-hours fasting insulin level, and the Homeostatic Model Assessment of Insulin Resistance (HOMA-IR). Insulin Resistance was diagnosed if the HOMA-IR was equal to or greater than 2.7 [13] using the formula: HOMA-IR = fasting insulin in mIU/L x fasting glucose in mg/dL/405.

## Statistical analysis

The baseline demographic and cardiometabolic characteristics were defined according to the HCV treatment regimen. They were tested for normal distribution using the Kolmogorov-Smirnov test. The continuous variables were presented as mean ± standard deviation (SD) and categorical variables were presented as proportions. The associations between baseline characteristics at follow-up were assessed using the chi-squared test for categorical variables and student t-test for continuous variables.

One-way analysis of variance (ANOVA) and chi-square tests were used to assess the distributions of numeric and categorical variables, respectively, across groups (before and after 12 weeks). Those characteristics statistically associated with the outcome of interest in the two groups after treatment were compared using a post-hoc test (LSD) for numeric variables and the chi-squared test for categorical variables to identify potential cardiometabolic risk factors associated with HCV treatment.

Wilks’ lambda multivariate test was used to evaluate the significance of the changes in studied parameters after HCV eradication and Levene’s test was used to assess data homogeneity. When changes were significant, and data were homogenous, general linear model (GLM) repeated measures tests and profile plots were conducted to compare these changes between the studied groups.

The power of sample size was estimated using g*power software 3.1.9.4, adjusted to a power of 80%, medium effect size, α error probability 0.05 for different variables. All analyses were done with 95% confidence intervals (95% CI) and considered a 2-tailed P value < 0.05 to be statistically significant. IBM SPSS software package version 21.0.0 was used for data analysis.

## Results

Four hundred patients with chronic HCV infection and midrange EF (40% to 49%) were included in this cross-sectional cohort study. The patients were divided into two groups according to their DAAs regimens and Child-Pugh classification. Group I (Child A) includes 200 patients who received (SOF+DCV) for twelve weeks. Group II (Child B) includes 200 patients who received (SOF+DCV+ RBV) for twelve weeks.

In our study 234 patients (58.5%) were men and 166 patients (41.5%) were women. Their mean age was 47.40 (±10.20) years. There was no significant difference between the patients in the two groups regarding age, gender incidence of hypertension, smoking, and NYHA Class: t = 1.227, P = 0.221 and X^2^ = 0.515, P = 0.473; X^2^ = 0.113, P = 0.203; X^2^ = 0.358, P = 0.550 and X^2^ = 2.918, P = 0.088 respectively. Also, there was no significant difference in either group regarding cardiac drug history, as shown in Table 1.

**Table 1:** Comparing basal demographic data and cardiac drug history of Group I and Group II.

	Group I	Group II	t/X^2^	P

**Age (Years)**	48.28 ± 11.89	46.51 ± 8.463	1.227^t^	0.221
**Males (%)**	112 (56%)	122 (61%)	0.515^X^	0.473
**Hypertension (%)**	163 (81.5%)	165 (82.5%)	0.113^X^	0.203
**Smoking (%)**	66 (33.0%)	60 (30.0%)	0.358^X^	0.550
NYHA I (%)*	166 (83%)	146 (73%)	2.918^X^	0.088
**Beta Blockers (%)**	174 (87%)	168 (84%)	1.337^X^	0.247
**ACEI/ARBS (%)**	184 (92%)	188 (94%)	0.915^X^	0.261
**Diuretics (%)**	96 (48%)	100 (50%)	0.824^X^	0.367
**Miniralocorticoids inhibitors (%)**	14 (7%)	16 (8%)	0.016^X^	0.942
**Digoxin (%)**	8 (4%)	10 (5%)	0.116^X^	0.733

* NYHA I and NYHA II only included. # Heart Rate (HR) < 60 b/min on resting ECG. t Independent-Samples T-test. X Chi-Square test. HTN hypertension.

The DAAs did not affect patients’ clinical, echocardiographic, or laboratory parameters of heart failure in either group, as measured by NYHH class, EF, and BNP, respectively, as shown in Table 2.

**Table 2:** Comparing NYHA Class, EF, and BNP in Groups I and II at Day 0 (baseline) and after three months (end of treatment).

		Group I – 0	Group I – 3	Group II – 0	Group II – 3	F/X^2^	P

**NYHA**	**I**	166 (83%)	162 (81%)	146 (73%)	144 (72%)	5.278^X^	0.153
	**II**	34 (17%)	38 (19%)	54 (27%)	56 (28%)		
**EF (%)**		45.66 ± 3.06	44.90 ± 4.43	44.75 ± 2.81	43.90 ± 2.84	3.439^F^	0.020^F^ 0.111^1^ 0.074^2^
**BNP (pg/ml)**		65.73 ± 16.68	69.83 ± 16.65	68.77 ± 17.42	72.67 ± 17.05	3.969^F^	0.011^F^ 0.063^1^ 0.071^2^

X Chi-Square test. F One – Way ANOVA test. ^1^ Post hoc test – least significant difference (LSD) between Group I – 0 and Group I – 3. ^2^ Post hoc test – least significant difference (LSD) between Group II – 0 and Group II – 3. NYHA New York Hear Association, EF; Ejection Fraction, BNP; Brain Natriuretic Peptide.

Also, DAAs in both groups did not produce any significant increase in the incidence of arrhythmias, with no significant difference in PVCs burden, highest and lowest heart, bradycardia, and non-sustained ventricular tachycardia (VT), as shown in Table 3.

**Table 3:** Comparing heart rate and induction of arrhythmia in Groups I and II at Day 0 (baseline) and after three months (end of treatment).

	Group A – 0	Group A – 3	Group B – 0	Group B – 3	F/X^2^	P

**Bradycardia (%)**^#^	0 (0%)	1 (0.5%)	0 (0%)	2 (1%)	2.010 ^X^	0.570 ^X^ 0.155^1^ 0.316^2^
**Non-Sustaned VT (%)**	1 (0.5%)	1 (0.5%)	1 (0.5%)	2 (1%)	0.608 ^X^	0.895^X^ 1.000^1^ 0.561^2^
**PVCs Burden %**	7.99 ± 4.31	8.88 ± 4.42	9.09 ± 4.41	9.14 ± 3.94	1.567^F^	0.197^F^ 0.142^1^ 0.934^2^
**Highest HR (b/min)**	93.35 ± 8.57	94.32 ± 8.64	92.07 ± 8.03	92.51 ± 7.31	1.481^F^	0.219^F^ 0.401^1^ 0.703^2^
**Lowest HR (b/min)**	63.35 ± 8.57	64.67 ± 8.88	62.77 ± 8.62	62.42 ± 7.79	1.363^F^	0.254^F^ 0.271^1^ 0.770^2^

# Heart Rate (HR) < 60 b/min on resting ECG. X Chi-Square test was used to compare all groups. F One – Way ANOVA test was used to compare all groups. ^1^ Comparison between Group A – 0 and Group A – 3. ^2^ Comparison between Group B – 0 and Group B – 3.

**Figure 1:**
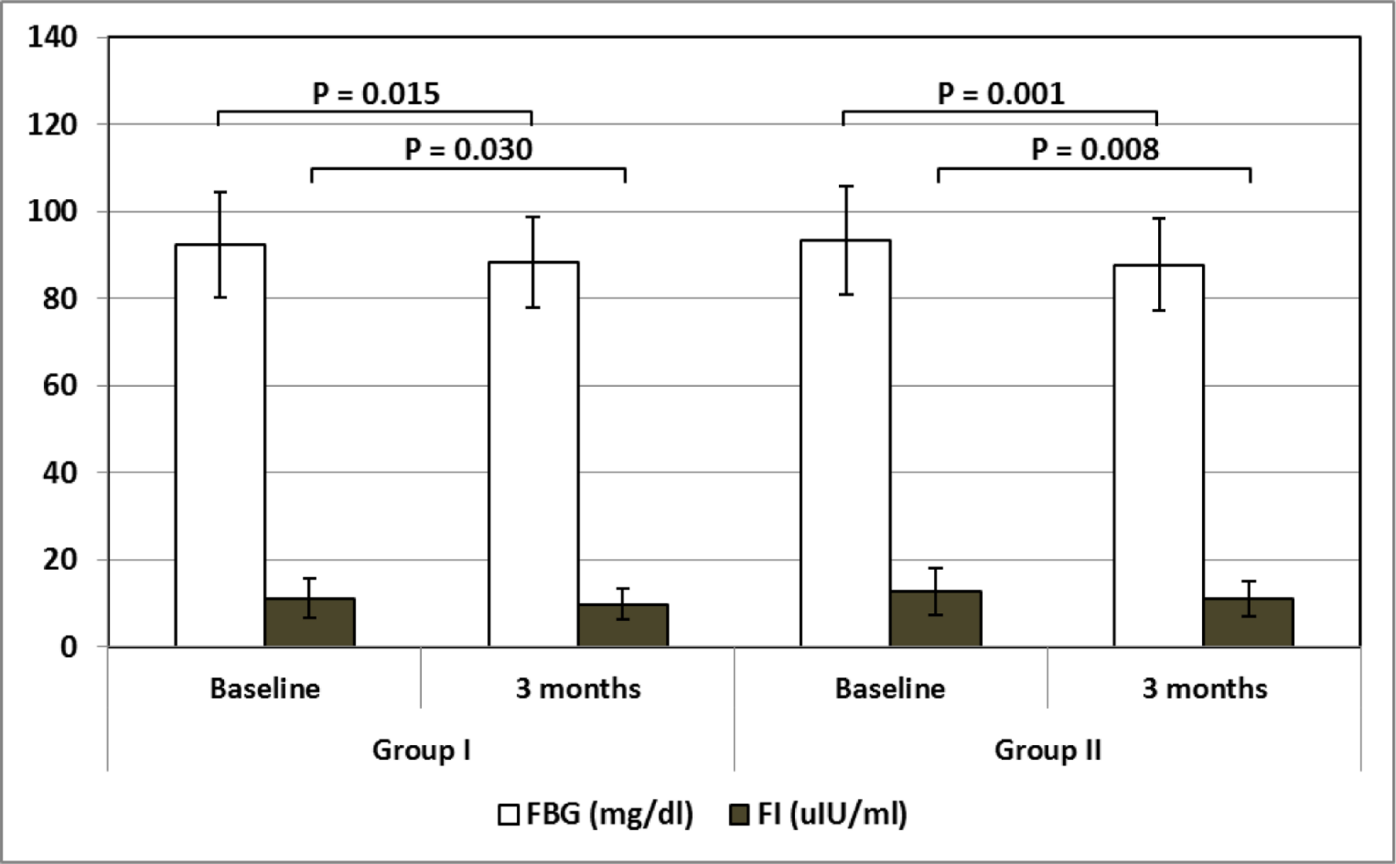
Fasting blood glucose and fasting insulin levels at baseline and after three months in Groups I and II. FBG: Fasting blood glucose; FI: Fasting insulin.

**Figure 2:**
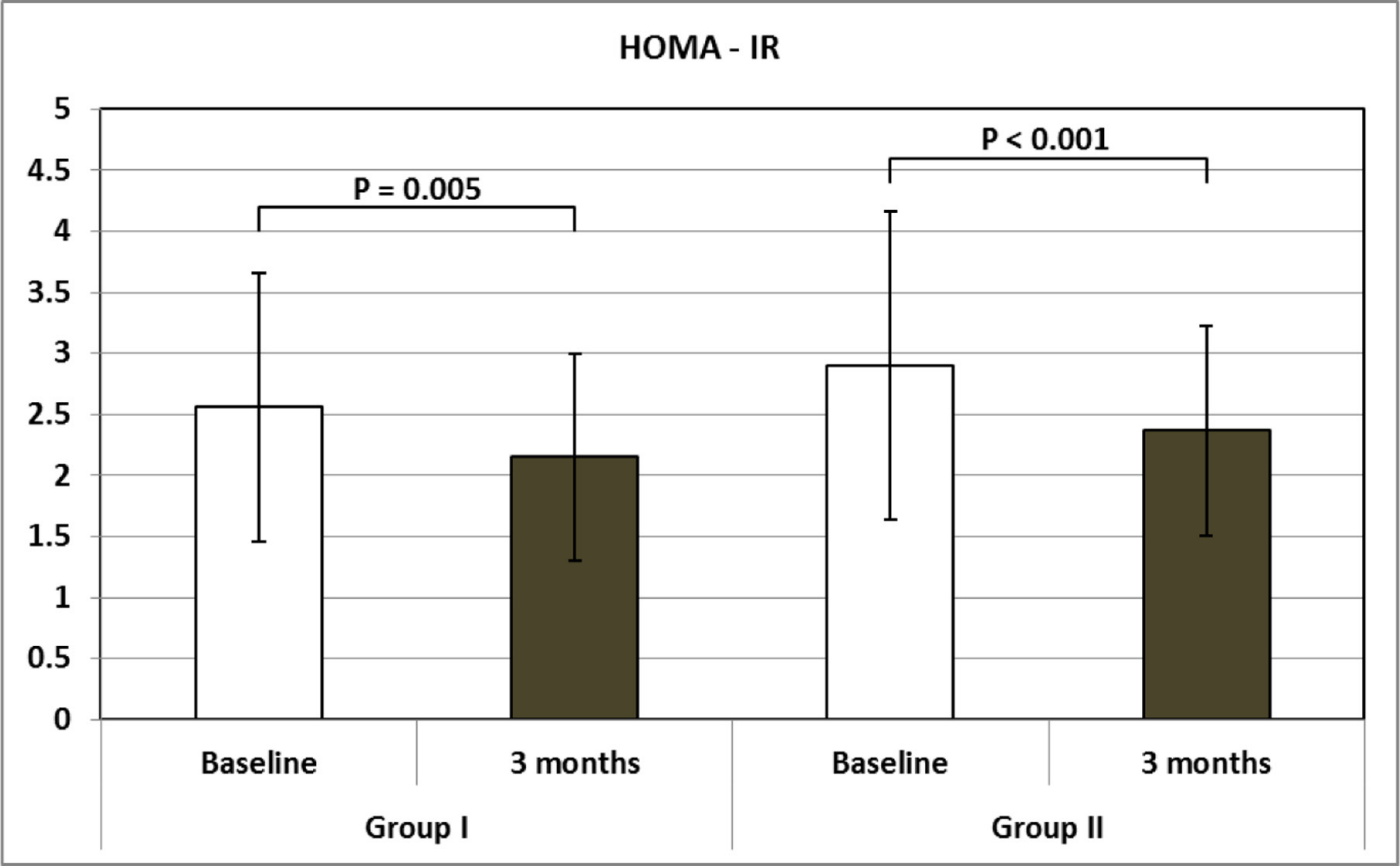
HOMA-IR index at baseline and after three months in Groups I and II. **HOMA – IR**: Homeostatic Model Assessment of Insulin Resistance.

There was a statistically significant decline in the FBG and improvement in insulin resistance after treatment in both groups, as shown in Table 4, Figures 1 and 2.

**Table 4:** Comparing fasting blood glucose, fasting insulin level, HOMA-IR and lipid profile in Groups I and II at Day 0 (baseline) and after three months (end of treatment).

	Group I – 0	Group I – 3	Group II – 0	Group II – 3	F	P

**FBG (mg/dl)**	92.30 ± 12.14	88.36 ± 10.27	93.38 ± 12.50	87.81 ± 10.47	5.998	0.001^F^ 0.015^1^ 0.001^2^
**FI (uIU/ml)**	11.18 ± 4.50	9.82 ± 3.61	12.65 ± 5.31	11.01 ± 3.98	6.960	<0.001^F^ 0.030^1^ 0.008^2^
**HOMA-IR**	2.56 ± 1.10	2.15 ± 0.85	2.90 ± 1.26	2.37 ± 0.86	9.414	<0.001^F^ 0.005^1^ <0.001^2^
**Chol (mg/dl)**	200.75 ± 45.16	213.19 ± 49.51	231.77 ± 41.59	239.34 ± 54.69	13.361	<0.001^F^ 0.068^1^ 0.265^2^
**LDL-C (mg/dl)**	127.14 ± 36.91	134.95 ± 43.59	129.16 ± 25.41	140.21 ± 29.63	2.867	0.036^F^ 0.109^1^ 0.028^2^
**HDL-C (mg/dl)**	44.69 ± 8.59	45.22 ± 7.65	37.06 ± 6.20	39.57 ± 7.51	27.794	<0.001^F^ 0.619^1^ 0.019^2^
**TGs (mg/dl)**	141.98 ± 45.72	151.98 ± 37.31	159.58 ± 58.03	166.39 ± 48.08	4.792	0.003^F^ 0.140^1^ 0.315^2^

F One – Way ANOVA test. ^1^ Post hoc test – least significant difference (LSD) between Group 1 – 0 and Group 1 – 3. ^2^ Post hoc test – least significant difference (LSD) between Group 2 – 0 and Group 2 – 3. FBG: Fasting Blood Glucose; FI: Fasting Insulin; HOMA-IR: Homeostatic Model Assessment of Insulin Resistance; Chol: Total Cholesterol; TGs: Triglycerides.

The general linear model (GLM) repeated measures tests were used to compare these changes in both groups. The FBG, FI and IR significantly declined after HCV eradication in both groups, Wilks’ lambda = 0.971, 0.899 and 0.902 respectively, F = 4.799, 22.184 and 21.503 respectively and P = 0.048, <0.001 and <0.001 respectively. However, when comparing these changes between both groups, there were no significant differences, as shown in Table 5 and Figure 4.

**Table 5:** Comparing the effects of DAAs regimens on metabolic parameters within and between studied groups after HCV eradication.

		Wilks’ Lambda	F	P

**FBG (mg/dl)**	Effect of DAAs regimens within groups	0.971	4.799	0.048
	Effect of DAAs regimens between groups	0.985	3.077	0.081
**FI (uIU/ml)**	Effect of DAAs regimens within groups	0.899	22.184	<0.001
	Effect of DAAs regimens between groups	0.995	1.032	0.311
**HOMA-IR**	Effect of DAAs regimens within groups	0.902	21.503	<0.001
	Effect of DAAs regimens between groups	0.986	2.821	0.095

FBG: Fasting Blood Glucose; FI: Fasting Insulin; HOMA-IR: Homeostatic Model Assessment of Insulin Resistance.

**Figure 3:**
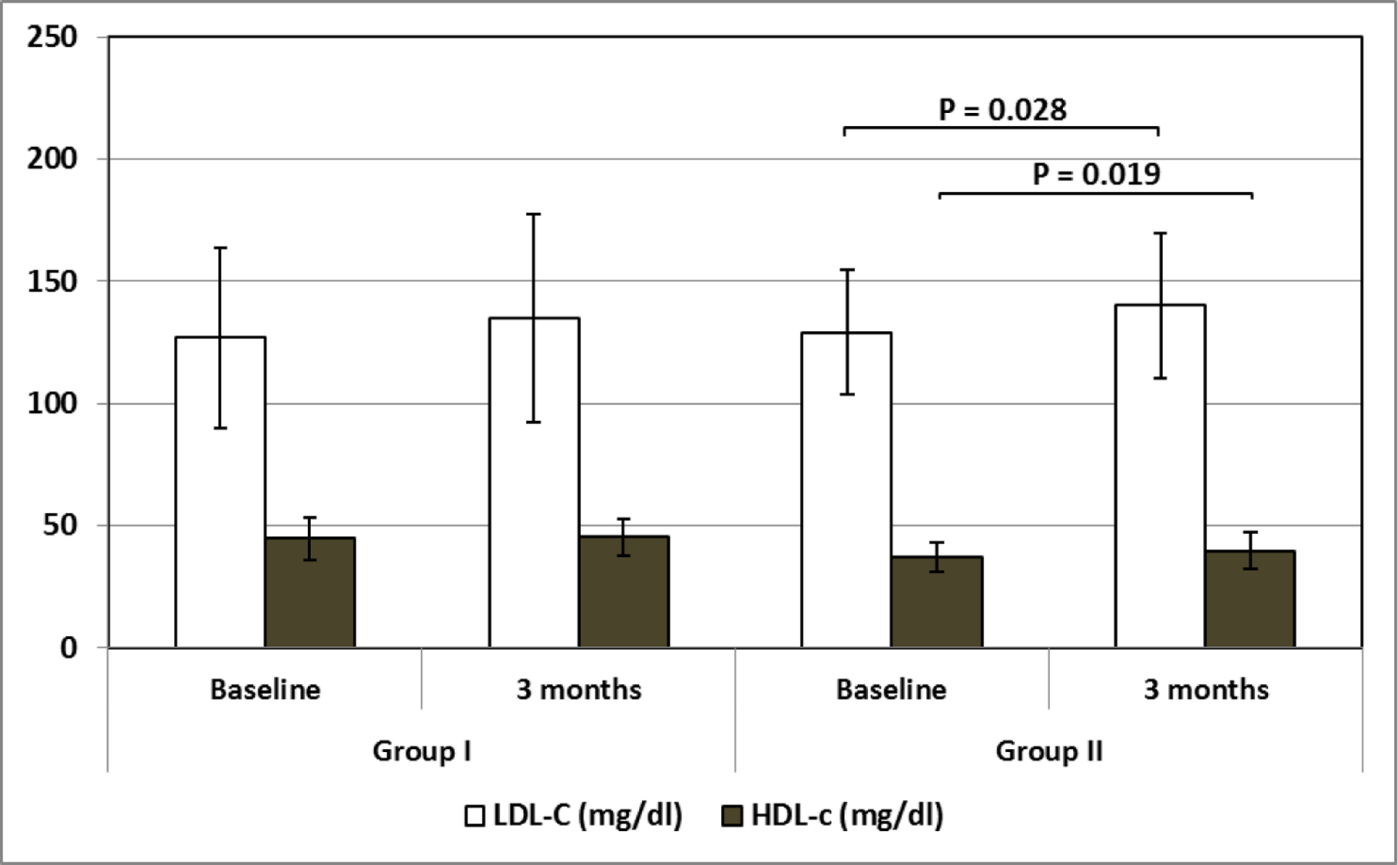
LDL-C and HDL-C levels at baseline and after three months in Groups I and II. **LDL-C**: Low-density lipoprotein cholesterol, **HDL-C**: High-density lipoprotein cholesterol.

In Group I, the total cholesterol, LDL-C, HDL-C, and triglycerides increased after treatment with no statistically significant difference, P = 0.068, 0.109, 0.619, and 0.140, respectively, as shown in Table 4 and Figure 3. In Group II, the LDL-C and HDL-C increased significantly after treatment, P = 0.028 and 0.019, respectively, as shown in Table 4 and Figure 3.

**Figure 4:**
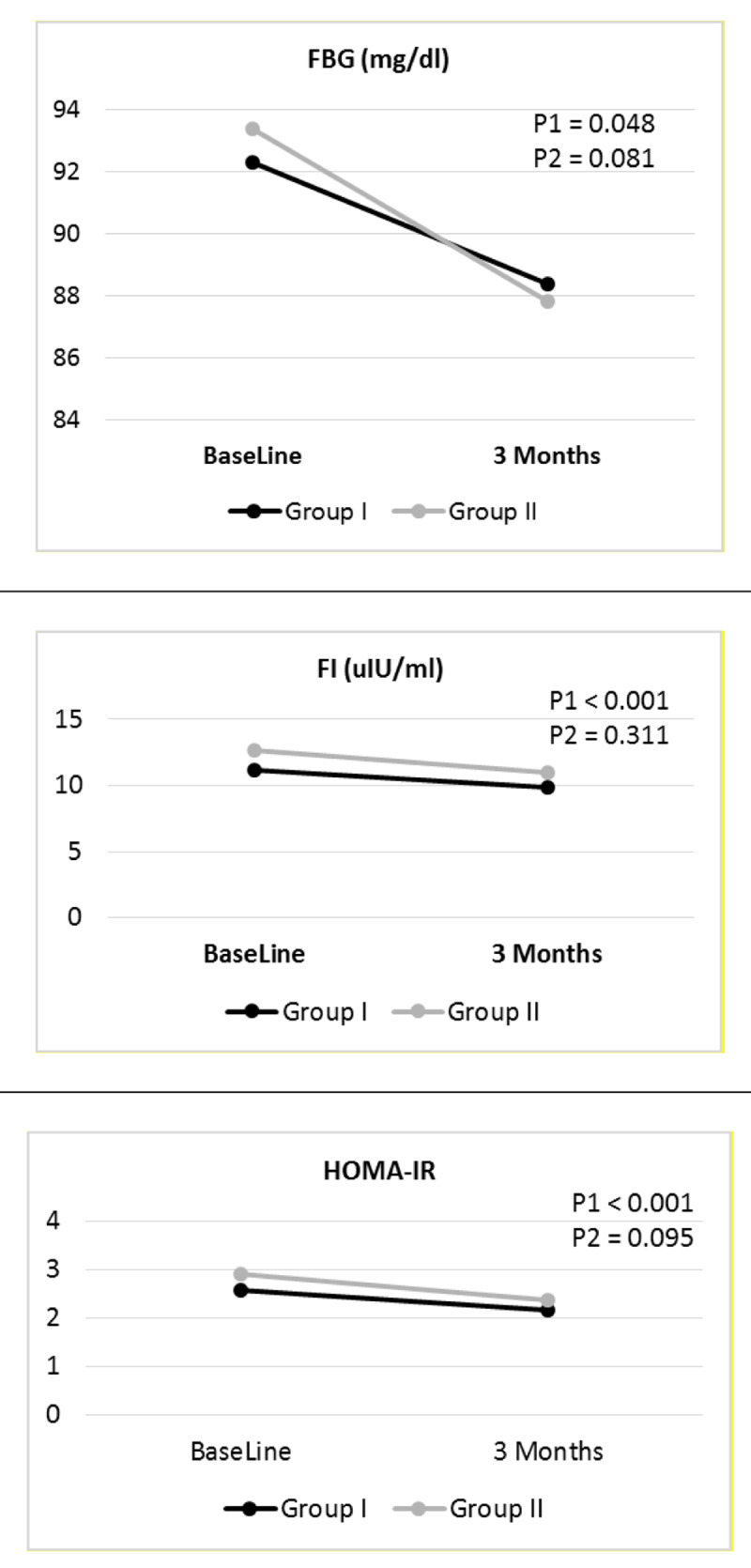
Changes in FBG, FI, and HOMA-IR levels at baseline and after three months in Groups I and II. **FBG**: Fasting Blood Glucose, **FI**: Fasting Insulin, **HOMA-IR**: Homeostatic Model Assessment of Insulin Resistance index, **P1**: Changes within groups, **P2**: Changes between groups.

## Discussion

Cardiovascular disorders and HCV infection have a high prevalence in the general population. Both have a high incidence in middle and old age populations. HCV infection was claimed to be a non-traditional risk factor for many cardiac conditions such as coronary artery disease, cardiomyopathies, and cardiac arrhythmias. Also, the heart could be affected by antiviral therapy [14].

The natural history of chronic HCV infection is characterized by developing several extra-hepatic manifestations that cause increased morbidity and mortality [15]. The wide variety of such manifestations has been defined as ‘HCV syndrome’ [16]. HCV syndrome denotes the clinical expression of the organic effects of HCV [17].

D’Agostino et al. [18] found that HCV infection surges cardiovascular hazards, including heart failure. Reda et al. [19] found that DAAs did not cause EF changes or new RWMAs in patients with normal baseline EF [14]. Our study found that DAAs in patients with midrange LVEF did not worsen their NYHA functional Class or EF by the end of treatment after three months. Also, there was a mild nonsignificant decrease in BNP. These results occurred in patients who received dual and triple DAAs. In our study, the DAAs did not produce any significant increase in the incidence of bradycardia, non-sustained VT, PVCs burden, or in highest and lowest heart rate.

We excluded the patients treated with amiodarone because DAAs may increase its concentration by inhibiting intestinal cytochrome P450 3A4 [20]. Similarly, a randomized study of 50 patients with structurally normal hearts treated with (SOF+ DCV) showed no significant effect on rhythm, heart rate, heart rate variability, or conductivity [21]. A meta-analysis including nearly 2300 HCV patients treated with SOF found that SOF was not associated with arrhythmias or significant bradycardias [22].

Some studies found a reduction of total cholesterol, decreased LDL levels, and a worsening of glucose metabolism in HCV infection [23,24]. Other studies described the impact of DAAs on glucose and lipid metabolism, and available data are incomplete and conflicting [25,26]. Hashimoto et al. [27] suggested that DAAs lead to a significant increase in LDL. This negative impact is absent in IFN-based therapy cases, probably due to the frequent presence of anorexia [28].

Badawi et al. [29] reported that serum levels of triglycerides, LDL-C total cholesterol, and ApoB were significantly lower in HCV patients. Also, Batsaikhan et al. [30] found that viral clearance was associated with a significantly increased triglycerides level at six months after treatment. However, this elevation was not detected in patients with non- sustained virological response (SVR). At the same time, Tran et al. [31] found improvement in serum triglycerides level, especially in patients with baseline elevation. They found a mean reduction of serum triglycerides by 28.6 mg/dl in all patients after eight weeks of treatment and reduction by 60.4 mg/dl in those who had baseline elevation. They also showed that 61% of patients with triglycerides elevation at baseline had levels below 175 mg/dl by the end of treatment.

In our data, the total cholesterol, HDL-C, LDL-C, and triglycerides increased after virus clearance. In this context, Khattab et al. [32] stated that the degree of changes in the lipid profile was affected by the degree of recovery of hepatic fibrosis. Its elevation after treatment is an indicator of the recovery of the liver cells and improved liver function. Moreover, the more advanced the hepatic affection before treatment, the more elevation occurs after viral clearance. The changes in the lipid profile are not a direct effect of the drug on lipid metabolism.

HCV utilizes the lipid metabolic pathways during replication leading to lipid profile changes [33]. It circulates in the blood within lipoproteins, known as lipoviroparticles. Lipoviroparticles, which utilize LDL-C, protect HCV from neutralization [34]. Also, Chronic hepatitis C is associated with metabolic complications like insulin resistance that may progress to type 2 diabetes, hepatic steatosis, and hypobetalipoproteinemia [35].

By the end of DAAs- based treatment, the HCV clearance leads to changes in peripheral and intrahepatic metabolic pathways [36]. The serum LDL-C surge levels likely reflect a shift in lipid metabolism due to inhibition of HCV replication [37].

In our study, the cure from HCV infection resulted in improved IR, which is consistent with the findings of Lim et al. and El Sagheer et al. [38,39]. Additionally, these findings mention that HCV infection treatment improved overall, hepatic, and adipose tissue insulin sensitivity.

The impact of HCV infection on IR could be explained in different ways, most of which involving insulin signaling [40,41,42]. The potential mechanisms include the degradation of insulin receptor substrate-1 (IRS-1), insulin activity inhibition, increased glucose synthesis, and release from hepatocytes [43] as well as hepatic inflammation and the production of pro-inflammatory cytokines (interleukin 8 & 18, tumor necrosis factor-alfa) increase IR in muscles and visceral fat [44]. Hence, HCV patients have a greater possibility of IR and type 2 diabetes mellitus than their matched non-HCV counterparts [45,46].

Finally, The metabolic changes in patients with HCV can affect their prognosis by modifying their cardiovascular risk factors independent of the direct virus effect on hepatic cells and the development of liver cirrhosis or hepatocellular carcinoma. However, the long-term effects of DAAs and virus clearance on metabolic profile and its clinical significance of these statistically significant changes, as shown in some studies, need to be followed over a more extended period.

## Conclusion

Both regimens of DAAs used in Egypt to treat patients with chronic HCV infection are safe in patients with NYHA functional Class I and II with midrange LVEF (40–49%). There are beneficial metabolic changes that occur with HCV clearance as an improvement of IR. On the other hand, attention should be paid to lipid profile changes, which can aggravate atherosclerosis and affect cardiac function negatively. Further research on a larger scale of heart failure patients and a more extended follow-up period is recommended.

## Study limitations

A relatively small number of patients were included in this cross-sectional study. Not included were diabetic patients, patients with severely reduced EF, nor patients with NYHA class III and IV. A more extended follow-up period may be addressed in further studies to assess the more prolonged effects of the DAAs. Finally, while this study looked at the systolic function, the effect of DAAs on other echocardiographic parameters may be tested in further studies.

## Data Accessibility Statements

The datasets used and or analyzed during the current study are available from the corresponding author on reasonable request.

## Figures and Tables

**Table:** 

2D	Two Dimensions
ALT	Alanine-Amino-Transferase
AST	Aspartate-Amino-Transferase
BNP	B-natriuretic peptide
CHF	Congestive Heart Failure
DAAs	Directly Acting Antiviral Agents
DCV	Daclatasvir
EDHS	Egyptian Demographic Health Survey
LVEF	Left Ventricular Ejection Fraction
FBG	Fasting Blood Glucose
HCV	Hepatitis C Virus
HDL-C	High-Density Lipoprotein – Cholesterol
HOMA-IR	Homeostatic Model Assessment of Insulin Resistance
INF	Interferon
INR	International Normalized Ratio
IR	Insulin Resistance
LDL-C	Low-Density Lipoprotein – Cholesterol
LV	Left Ventricle
NCCVH	National Committee for Control of Viral Hepatitis
NYHA	New York Heart Association
PCR	Polymerase Chain Reaction
PT	Prothrombin Time
RBV	Ribavirin
RNA	Ribonucleic Acid
RWMAs	Regional Wall Motion Abnormalities
SOF	Sofosbuvir
SVR	Sustained Virological Response
VT	Ventricular Tachycardia
